# *catena*-Poly[[[pentaaquaeuropium(III)]-μ-5-sulfonatoisophthalato-κ^4^ *O* ^1^,*O* ^1′^:*O* ^3^,*O* ^3′^] 4,4’-bipyridine *N*,*N*′-dioxide hemisolvate trihydrate]

**DOI:** 10.1107/S1600536810041838

**Published:** 2010-10-23

**Authors:** Ai-Zhi Wu, Seik Weng Ng

**Affiliations:** a School of Chinese Materia Medica, Guangzhou University of Chinese Medicine, Guangzhou 510006, People’s Republic of China; b Department of Chemistry, University of Malaya, 50603 Kuala Lumpur, Malaysia

## Abstract

In the crystal structure of the title compound, {[Eu(C_8_H_3_O_7_S)(H_2_O)_5_]·0.5C_10_H_8_N_2_O_2_·3H_2_O}_*n*_, the Eu^III^ coordination polymer displays a ribbon motif as the 5-sulfoisopthalate anion uses one of carboxyl –CO_2_ units to chelate to a Eu atom and the other to bind to other two Eu atoms; the sulfonyl –SO_3_ unit is not involved in coordination. Adjacent ribbons are linked by O—H⋯O hydrogen bonds, generating a three-dimensional network. The 4,4′-bipyridine-*N*,*N*′-dioxide mol­ecule lies on an inversion centre and is hydrogen-bonded to the complex network. The coordination geometry of the Eu atom is a monocapped square anti­prism.

## Related literature

For a related structure, see: Hu *et al.* (2005 ▶).
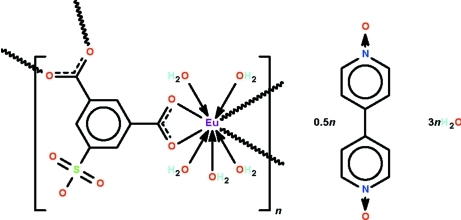


## Experimental

### 

#### Crystal data

[Eu(C_8_H_3_O_7_S)(H_2_O)_5_]·0.5C_10_H_8_N_2_O_2_·3H_2_O
*M* *_r_* = 633.34
Triclinic, 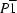

*a* = 10.7800 (4) Å
*b* = 10.8978 (3) Å
*c* = 11.4048 (3) Å
α = 89.383 (2)°
β = 62.989 (1)°
γ = 62.678 (6)°
*V* = 1026.90 (9) Å^3^
*Z* = 2
Mo *K*α radiation
μ = 3.24 mm^−1^
*T* = 293 K
0.25 × 0.20 × 0.15 mm

#### Data collection

Rigaku R-AXIS RAPID diffractometer
Absorption correction: multi-scan (*ABSCOR*; Higashi, 1995 ▶) *T*_min_ = 0.631, *T*_max_ = 1.000
10108 measured reflections
4658 independent reflections
4509 reflections with *I* > 2σ(*I*)
*R*_int_ = 0.033

#### Refinement

*R*[*F* ^2^ > 2σ(*F* ^2^)] = 0.032
*wR*(*F* ^2^) = 0.091
*S* = 1.20
4658 reflections
289 parameters
H-atom parameters constrained
Δρ_max_ = 3.40 e Å^−3^
Δρ_min_ = −1.13 e Å^−3^

### 

Data collection: *RAPID-AUTO* (Rigaku, 1998 ▶); cell refinement: *RAPID-AUTO*; data reduction: *CrystalStructure* (Rigaku/MSC, 2002 ▶); program(s) used to solve structure: *SHELXS97* (Sheldrick, 2008 ▶); program(s) used to refine structure: *SHELXL97* (Sheldrick, 2008 ▶); molecular graphics: *X-SEED* (Barbour, 2001 ▶); software used to prepare material for publication: *publCIF* (Westrip, 2010 ▶).

## Supplementary Material

Crystal structure: contains datablocks global, I. DOI: 10.1107/S1600536810041838/xu5055sup1.cif

Structure factors: contains datablocks I. DOI: 10.1107/S1600536810041838/xu5055Isup2.hkl

Additional supplementary materials:  crystallographic information; 3D view; checkCIF report

## Figures and Tables

**Table 1:** Hydrogen-bond geometry (Å, °)

*D*—H⋯*A*	*D*—H	H⋯*A*	*D*⋯*A*	*D*—H⋯*A*
O1w—H11⋯O8	0.84	1.87	2.673 (4)	159
O1w—H12⋯O6w	0.84	1.91	2.710 (4)	160
O2w—H22⋯O1^i^	0.84	2.11	2.804 (4)	140
O2w—H21⋯O3w^i^	0.84	2.46	3.148 (5)	140
O3w—H31⋯O7w	0.84	1.91	2.742 (4)	175
O3w—H32⋯O8w	0.84	1.87	2.704 (4)	172
O4w—H41⋯O8^ii^	0.83	1.82	2.648 (4)	171
O4w—H42⋯O5^iii^	0.83	2.02	2.840 (4)	166
O5w—H51⋯O7w	0.83	2.30	3.026 (5)	146
O6w—H62⋯O6^iv^	0.85	2.18	2.955 (5)	151
O6w—H61⋯O6^v^	0.85	2.18	2.807 (5)	130
O7w—H71⋯O1^i^	0.84	2.49	3.191 (4)	142
O7w—H72⋯O7^vi^	0.84	2.03	2.860 (5)	169
O8w—H81⋯O2^vii^	0.85	2.17	2.867 (4)	140
O8w—H82⋯O5^viii^	0.85	2.07	2.879 (5)	159

Symmetry codes: (i) 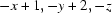; (ii) 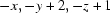; (iii) 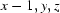; (iv) 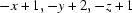; (v) 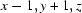; (vi) 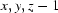; (vii) 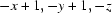; (viii) 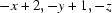.

**Fig. 1.**
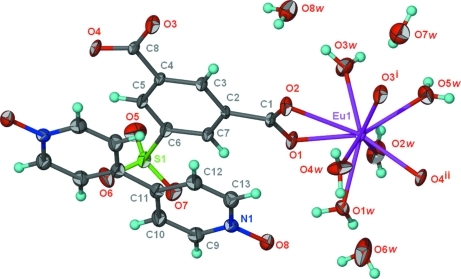
Thermal ellipsoid plot (Barbour, 2001) of a portion of the polymeric structure of Eu(H2O)5(C8H3O7S).3H2O.0.5C10H8N2O2 at the 50% probability level; hydrogen atoms are drawn as spheres of arbitrary radius. The 4,4'-bipyridine N-oxide molecule lies on a center-of-inversion.

**Fig. 2.**
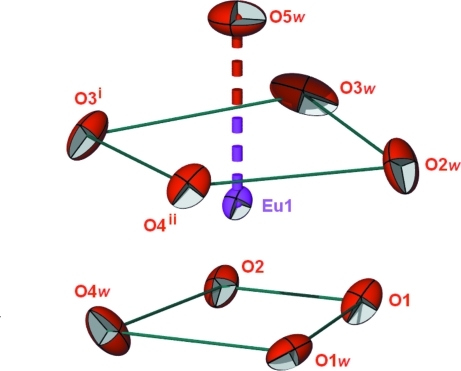
Nine-coordinate geometry of Eu.

**Fig. 3.**
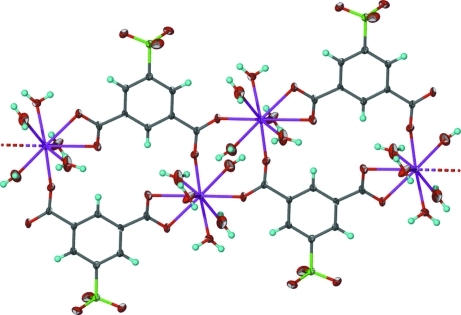
Ribbon structure.

## supplementary crystallographic information

### Comment

In the crystal structure of pentaqua(5-sulfoisophthalato)europium monohydrate sesqui-4,4'-bipyridine, the 5-sulfoisophthalate trianion *O*,*O*'-chelates to two metal atoms to furnish a polymeric chain structure. The chains are linked by hydrogen bonds into a three-dimensional network, and the *N*-heterocycles occupy the cavities of the network. The geometry of the metal atom is a monocapped square-antiprism (Hu *et al.*, 2005). A similar hydrothermal synthesis with 4,4'-bipyridine *N*-oxide in place of 4,4'-bipyridine affords the corresponding co-crystal, but the co-crystal is a trihydrated1:0.5 co-crystal. In Eu(H_2_O)_5_(C_8_H_3_O_7_S)^.^3H_2_O^.^0.5C_10_H_8_N_2_O_2_ (Scheme I), the 5-sulfoisopthalate group uses one of carboxyl –CO_2_ units to chelate to a Eu(III) atom and uses the other to bind to two other metal atoms; the sulfonyl –SO_3_ unit is not involved in coordination (Fig. 1). The geometry of the europium atom is a monocapped square-antiprism (Fig. 2). Adjacent ribbons (Fig. 3) are linked by O–H···O hydrogen bonds to generate a three-dimensional network (Table 3). The 4,4'-bipyridine *N*-oxide molecule, which lies on a center-of-inversion, is hydrogen-bonded to the network.

### Experimental

Europium nitrate hexahydrate (0.22 g, 0.5 mmol), monosodium 5-sulfoisophthalate (0.16 g, 0.5 mmol) and 4,4'-bipyridine-N,N'-dioxide (0.095 g, 0.5 mmol) were placed in a 25-ml Teflon-lined, stainless-steel Parr bomb along with water (15 ml). The bomb was heated to 433 K for 72 h. It was then cooled down to room temperature at a rate of 5 K an hour. Colorless crystals were collected and washed with water (0.195 g, yield 55% based on Eu). The product is stable in air and and is insoluble in water and common organic solvents. CH&N elemental analysis. Calc. for C_18_H_25_N_2_EuO_16_S (709.42): C 34.45, H 3.55, N 3.97%. Found: C 34.24, H 3.50, N 4.08%.

### Refinement

Carbon-bound hydrogen atoms were placed in calculated positions (C–H 0.93 Å, *U*_iso_(H) 1.2 times *U*_eq_(C), and were included in the refinement in the riding model approximation. The water H-atoms were placed placed in calculated positions (O–H 0.84 Å and H···H 1.37 Å) on the basis of hydrogen bonding; their temperature factors were tied by a factor of 1.5.

The final difference Fourier map had a peak and a hole in the vicinity of Eu1, but was otherwise featureless. Attempts to decrease their magnitudes by lowering the 2-theta limit did not lower these values, and the structure did not exhibit any signs of twinning.

### Crystal data

**Table:**

[Eu(C_8_H_3_O_7_S)(H_2_O)_5_]·0.5C_10_H_8_N_2_O_2_·3H_2_O	*Z* = 2
*M**_r_* = 633.34	*F*(000) = 630
Triclinic, *P*1	*D*_x_ = 2.048 Mg m^−^^3^
Hall symbol: -P 1	Mo *K*α radiation, λ = 0.71073 Å
*a* = 10.7800 (4) Å	Cell parameters from 9807 reflections
*b* = 10.8978 (3) Å	θ = 3.5–27.5°
*c* = 11.4048 (3) Å	µ = 3.24 mm^−^^1^
α = 89.383 (2)°	*T* = 293 K
β = 62.989 (1)°	Prism, colorless
γ = 62.678 (6)°	0.25 × 0.20 × 0.15 mm
*V* = 1026.90 (9) Å^3^	

### Data collection

**Table:**

Rigaku R-AXIS RAPID diffractometer	4658 independent reflections
Radiation source: fine-focus sealed tube	4509 reflections with *I* > 2σ(*I*)
graphite	*R*_int_ = 0.033
Detector resolution: 10.000 pixels mm^-1^	θ_max_ = 27.5°, θ_min_ = 3.5°
ω scans	*h* = −13→13
Absorption correction: multi-scan (*ABSCOR*; Higashi, 1995)	*k* = −14→14
*T*_min_ = 0.631, *T*_max_ = 1.000	*l* = −14→14
10108 measured reflections	

### Refinement

**Table:**

Refinement on *F*^2^	Primary atom site location: structure-invariant direct methods
Least-squares matrix: full	Secondary atom site location: difference Fourier map
*R*[*F*^2^ > 2σ(*F*^2^)] = 0.032	Hydrogen site location: inferred from neighbouring sites
*wR*(*F*^2^) = 0.091	H-atom parameters constrained
*S* = 1.20	*w* = 1/[σ^2^(*F*_o_^2^) + (0.0569*P*)^2^ + 0.4859*P*] where *P* = (*F*_o_^2^ + 2*F*_c_^2^)/3
4658 reflections	(Δ/σ)_max_ = 0.001
289 parameters	Δρ_max_ = 3.40 e Å^−^^3^
0 restraints	Δρ_min_ = −1.13 e Å^−^^3^

### Fractional atomic coordinates and isotropic or equivalent isotropic displacement parameters (Å^2^)

**Table:**

	*x*	*y*	*z*	*U*_iso_*/*U*_eq_	
Eu1	0.222361 (16)	0.944055 (13)	0.073400 (13)	0.01756 (8)	
S1	0.84263 (11)	0.67200 (9)	0.32120 (9)	0.02471 (18)	
O1	0.4314 (3)	0.8569 (3)	0.1429 (3)	0.0305 (6)	
O2	0.3992 (3)	0.6830 (3)	0.0953 (3)	0.0285 (5)	
O3	0.8412 (4)	0.1844 (3)	0.0243 (3)	0.0346 (6)	
O4	0.9706 (3)	0.1556 (3)	0.1368 (3)	0.0295 (6)	
O5	1.0036 (4)	0.6411 (4)	0.2226 (3)	0.0418 (7)	
O6	0.8400 (4)	0.6030 (3)	0.4321 (3)	0.0396 (7)	
O7	0.7229 (4)	0.8236 (3)	0.3736 (3)	0.0375 (6)	
O8	0.1731 (4)	0.9846 (3)	0.4955 (3)	0.0329 (6)	
O1W	0.1051 (3)	1.0804 (3)	0.3032 (3)	0.0310 (6)	
H11	0.1453	1.0318	0.3474	0.047*	
H12	0.0801	1.1650	0.3258	0.047*	
O2W	0.3042 (4)	1.1235 (3)	0.0498 (4)	0.0410 (7)	
H21	0.3387	1.1230	0.1028	0.061*	
H22	0.3771	1.1045	−0.0306	0.061*	
O3W	0.4909 (4)	0.8044 (3)	−0.1330 (3)	0.0506 (9)	
H31	0.5216	0.8429	−0.1957	0.076*	
H32	0.5598	0.7170	−0.1571	0.076*	
O4W	0.0462 (4)	0.8763 (3)	0.2470 (3)	0.0410 (7)	
H41	−0.0306	0.9237	0.3254	0.061*	
H42	0.0447	0.8044	0.2251	0.061*	
O5W	0.2265 (4)	1.0292 (4)	−0.1370 (3)	0.0441 (8)	
H51	0.3014	1.0388	−0.1953	0.066*	
H52	0.1451	1.0708	−0.1456	0.066*	
O6W	0.1006 (6)	1.3246 (4)	0.3568 (5)	0.0661 (12)	
H61	0.0007	1.3802	0.4141	0.099*	
H62	0.1513	1.3186	0.3980	0.099*	
O7W	0.5761 (4)	0.9318 (4)	−0.3410 (3)	0.0442 (8)	
H71	0.6177	0.9731	−0.3246	0.066*	
H72	0.6113	0.9114	−0.4245	0.066*	
O8W	0.6904 (4)	0.5181 (3)	−0.2118 (3)	0.0423 (7)	
H81	0.6327	0.4968	−0.1455	0.064*	
H82	0.7768	0.4929	−0.2110	0.064*	
N1	0.2607 (4)	0.8511 (3)	0.4986 (3)	0.0260 (6)	
C1	0.4710 (4)	0.7263 (4)	0.1275 (3)	0.0211 (6)	
C2	0.6071 (4)	0.6265 (3)	0.1492 (3)	0.0182 (6)	
C3	0.6773 (4)	0.4793 (3)	0.1120 (3)	0.0199 (6)	
H3	0.6427	0.4402	0.0698	0.024*	
C4	0.7996 (4)	0.3904 (3)	0.1383 (3)	0.0185 (6)	
C5	0.8531 (4)	0.4479 (3)	0.1990 (3)	0.0216 (6)	
H5	0.9366	0.3882	0.2142	0.026*	
C6	0.7815 (4)	0.5957 (3)	0.2375 (4)	0.0217 (6)	
C7	0.6609 (4)	0.6842 (3)	0.2107 (3)	0.0203 (6)	
H7	0.6157	0.7825	0.2338	0.024*	
C8	0.8774 (4)	0.2302 (3)	0.0962 (3)	0.0201 (6)	
C9	0.3563 (5)	0.8243 (4)	0.5518 (4)	0.0305 (8)	
H9	0.3592	0.8988	0.5872	0.037*	
C10	0.4495 (5)	0.6877 (4)	0.5543 (4)	0.0320 (8)	
H10	0.5150	0.6705	0.5916	0.038*	
C11	0.4475 (4)	0.5746 (4)	0.5022 (3)	0.0248 (7)	
C12	0.3437 (5)	0.6074 (4)	0.4505 (4)	0.0292 (7)	
H12A	0.3361	0.5351	0.4174	0.035*	
C13	0.2525 (5)	0.7454 (4)	0.4480 (4)	0.0311 (8)	
H13	0.1856	0.7657	0.4116	0.037*	

### Atomic displacement parameters (Å^2^)

**Table:**

	*U*^11^	*U*^22^	*U*^33^	*U*^12^	*U*^13^	*U*^23^
Eu1	0.01783 (11)	0.01261 (11)	0.02442 (12)	−0.00607 (8)	−0.01399 (8)	0.00504 (7)
S1	0.0289 (4)	0.0182 (4)	0.0365 (4)	−0.0127 (3)	−0.0228 (4)	0.0062 (3)
O1	0.0349 (14)	0.0155 (11)	0.0467 (15)	−0.0070 (11)	−0.0306 (13)	0.0079 (11)
O2	0.0316 (14)	0.0250 (12)	0.0428 (14)	−0.0151 (11)	−0.0283 (12)	0.0145 (11)
O3	0.0466 (17)	0.0176 (11)	0.0531 (17)	−0.0123 (12)	−0.0389 (14)	0.0047 (11)
O4	0.0291 (13)	0.0154 (10)	0.0412 (14)	−0.0024 (10)	−0.0248 (12)	0.0052 (10)
O5	0.0353 (16)	0.0459 (17)	0.0472 (17)	−0.0255 (14)	−0.0181 (14)	0.0017 (14)
O6	0.063 (2)	0.0370 (15)	0.0425 (15)	−0.0283 (15)	−0.0416 (15)	0.0158 (13)
O7	0.0460 (17)	0.0197 (12)	0.0515 (17)	−0.0116 (12)	−0.0333 (15)	0.0025 (12)
O8	0.0399 (15)	0.0218 (12)	0.0321 (13)	−0.0094 (12)	−0.0210 (12)	0.0075 (11)
O1W	0.0447 (16)	0.0234 (12)	0.0295 (12)	−0.0136 (11)	−0.0260 (12)	0.0065 (10)
O2W	0.0412 (17)	0.0359 (15)	0.064 (2)	−0.0283 (14)	−0.0311 (16)	0.0240 (15)
O3W	0.0385 (17)	0.0373 (16)	0.0340 (15)	−0.0015 (14)	−0.0059 (13)	0.0146 (13)
O4W	0.0450 (17)	0.0279 (13)	0.0320 (13)	−0.0247 (13)	0.0001 (12)	−0.0031 (11)
O5W	0.0452 (18)	0.0478 (18)	0.0304 (14)	−0.0162 (15)	−0.0202 (13)	0.0166 (14)
O6W	0.085 (3)	0.0321 (17)	0.117 (4)	−0.0247 (19)	−0.082 (3)	0.021 (2)
O7W	0.0430 (18)	0.0456 (18)	0.0454 (18)	−0.0269 (16)	−0.0190 (15)	0.0187 (15)
O8W	0.0333 (15)	0.0390 (16)	0.0533 (18)	−0.0186 (13)	−0.0208 (14)	0.0149 (14)
N1	0.0280 (15)	0.0233 (14)	0.0203 (12)	−0.0103 (12)	−0.0103 (11)	0.0060 (11)
C1	0.0186 (15)	0.0184 (15)	0.0246 (15)	−0.0064 (13)	−0.0130 (13)	0.0089 (13)
C2	0.0204 (14)	0.0155 (14)	0.0199 (13)	−0.0065 (12)	−0.0139 (12)	0.0065 (11)
C3	0.0219 (15)	0.0166 (13)	0.0249 (14)	−0.0090 (12)	−0.0153 (13)	0.0064 (13)
C4	0.0199 (14)	0.0122 (12)	0.0233 (14)	−0.0060 (11)	−0.0131 (12)	0.0038 (11)
C5	0.0213 (15)	0.0163 (14)	0.0290 (15)	−0.0057 (12)	−0.0180 (13)	0.0046 (12)
C6	0.0234 (16)	0.0157 (14)	0.0314 (16)	−0.0099 (13)	−0.0179 (14)	0.0051 (12)
C7	0.0231 (15)	0.0134 (13)	0.0269 (15)	−0.0080 (12)	−0.0157 (13)	0.0054 (12)
C8	0.0193 (14)	0.0132 (13)	0.0257 (15)	−0.0051 (12)	−0.0131 (12)	0.0030 (12)
C9	0.035 (2)	0.0258 (17)	0.0312 (17)	−0.0146 (16)	−0.0185 (16)	0.0045 (15)
C10	0.036 (2)	0.0304 (18)	0.0378 (19)	−0.0156 (16)	−0.0256 (17)	0.0089 (16)
C11	0.0223 (16)	0.0251 (17)	0.0244 (15)	−0.0126 (14)	−0.0095 (13)	0.0096 (13)
C12	0.0340 (19)	0.0270 (17)	0.0312 (17)	−0.0159 (16)	−0.0194 (16)	0.0062 (15)
C13	0.0314 (19)	0.0312 (18)	0.0351 (18)	−0.0151 (16)	−0.0210 (16)	0.0075 (15)

### Geometric parameters (Å, °)

**Table:**

Eu1—O1	2.500 (2)	O6W—H61	0.8513
Eu1—O2	2.688 (2)	O6W—H62	0.8503
Eu1—O3^i^	2.298 (3)	O7W—H71	0.8376
Eu1—O4^ii^	2.390 (2)	O7W—H72	0.8365
Eu1—O1W	2.455 (2)	O8W—H81	0.8459
Eu1—O2W	2.456 (3)	O8W—H82	0.8457
Eu1—O3W	2.466 (3)	N1—C9	1.343 (5)
Eu1—O4W	2.431 (3)	N1—C13	1.350 (5)
Eu1—O5W	2.552 (3)	C1—C2	1.502 (4)
S1—O5	1.445 (3)	C2—C7	1.391 (4)
S1—O7	1.454 (3)	C2—C3	1.392 (4)
S1—O6	1.460 (3)	C3—C4	1.395 (4)
S1—C6	1.769 (3)	C3—H3	0.9300
O1—C1	1.273 (4)	C4—C5	1.381 (4)
O2—C1	1.246 (4)	C4—C8	1.513 (4)
O3—C8	1.245 (4)	C5—C6	1.396 (4)
O4—C8	1.245 (4)	C5—H5	0.9300
O8—N1	1.333 (4)	C6—C7	1.385 (4)
O1W—H11	0.8386	C7—H7	0.9300
O1W—H12	0.8384	C9—C10	1.371 (5)
O2W—H21	0.8400	C9—H9	0.9300
O2W—H22	0.8380	C10—C11	1.387 (5)
O3W—H31	0.8383	C10—H10	0.9300
O3W—H32	0.8396	C11—C12	1.398 (5)
O4W—H41	0.8345	C11—C11^iii^	1.479 (7)
O4W—H42	0.8346	C12—C13	1.378 (5)
O5W—H51	0.8333	C12—H12A	0.9300
O5W—H52	0.8349	C13—H13	0.9300
			
O3^i^—Eu1—O4^ii^	90.47 (9)	Eu1—O3W—H32	130.2
O3^i^—Eu1—O4W	70.06 (11)	H31—O3W—H32	109.5
O4^ii^—Eu1—O4W	82.34 (10)	Eu1—O4W—H41	129.1
O3^i^—Eu1—O2W	144.73 (11)	Eu1—O4W—H42	119.2
O4^ii^—Eu1—O2W	79.72 (10)	H41—O4W—H42	110.4
O4W—Eu1—O2W	140.17 (11)	Eu1—O5W—H51	123.4
O3^i^—Eu1—O1W	135.51 (11)	Eu1—O5W—H52	124.1
O4^ii^—Eu1—O1W	70.13 (9)	H51—O5W—H52	110.6
O4W—Eu1—O1W	67.97 (10)	H61—O6W—H62	106.3
O2W—Eu1—O1W	72.54 (11)	H71—O7W—H72	110.2
O3^i^—Eu1—O3W	82.80 (13)	H81—O8W—H82	107.4
O4^ii^—Eu1—O3W	137.24 (10)	O8—N1—C9	119.1 (3)
O4W—Eu1—O3W	132.75 (10)	O8—N1—C13	119.7 (3)
O2W—Eu1—O3W	81.93 (12)	C9—N1—C13	121.2 (3)
O1W—Eu1—O3W	138.25 (12)	O2—C1—O1	121.0 (3)
O3^i^—Eu1—O1	129.26 (8)	O2—C1—C2	121.4 (3)
O4^ii^—Eu1—O1	137.26 (9)	O1—C1—C2	117.6 (3)
O4W—Eu1—O1	95.95 (11)	C7—C2—C3	119.8 (3)
O2W—Eu1—O1	74.72 (9)	C7—C2—C1	118.7 (3)
O1W—Eu1—O1	69.79 (9)	C3—C2—C1	121.5 (3)
O3W—Eu1—O1	71.94 (11)	C2—C3—C4	119.8 (3)
O3^i^—Eu1—O5W	72.39 (11)	C2—C3—H3	120.1
O4^ii^—Eu1—O5W	69.93 (10)	C4—C3—H3	120.1
O4W—Eu1—O5W	132.53 (12)	C5—C4—C3	120.3 (3)
O2W—Eu1—O5W	72.42 (11)	C5—C4—C8	119.9 (3)
O1W—Eu1—O5W	130.36 (10)	C3—C4—C8	119.8 (3)
O3W—Eu1—O5W	67.78 (10)	C4—C5—C6	119.9 (3)
O1—Eu1—O5W	130.58 (11)	C4—C5—H5	120.1
O3^i^—Eu1—O2	80.16 (8)	C6—C5—H5	120.1
O4^ii^—Eu1—O2	152.27 (9)	C7—C6—C5	120.0 (3)
O4W—Eu1—O2	69.93 (10)	C7—C6—S1	119.3 (2)
O2W—Eu1—O2	122.10 (9)	C5—C6—S1	120.7 (3)
O1W—Eu1—O2	98.56 (8)	C6—C7—C2	120.2 (3)
O3W—Eu1—O2	67.78 (9)	C6—C7—H7	119.9
O1—Eu1—O2	49.82 (8)	C2—C7—H7	119.9
O5W—Eu1—O2	129.85 (10)	O3—C8—O4	125.6 (3)
O5—S1—O7	113.70 (19)	O3—C8—C4	116.8 (3)
O5—S1—O6	112.2 (2)	O4—C8—C4	117.7 (3)
O7—S1—O6	110.88 (19)	N1—C9—C10	120.3 (4)
O5—S1—C6	107.16 (17)	N1—C9—H9	119.8
O7—S1—C6	106.77 (16)	C10—C9—H9	119.8
O6—S1—C6	105.62 (16)	C9—C10—C11	121.0 (3)
C1—O1—Eu1	98.7 (2)	C9—C10—H10	119.5
C1—O2—Eu1	90.40 (19)	C11—C10—H10	119.5
C8—O3—Eu1^i^	164.5 (2)	C10—C11—C12	116.8 (3)
C8—O4—Eu1^iv^	144.6 (2)	C10—C11—C11^iii^	122.1 (4)
Eu1—O1W—H11	113.1	C12—C11—C11^iii^	121.0 (4)
Eu1—O1W—H12	126.6	C13—C12—C11	120.9 (3)
H11—O1W—H12	109.5	C13—C12—H12A	119.5
Eu1—O2W—H21	109.7	C11—C12—H12A	119.5
Eu1—O2W—H22	109.1	N1—C13—C12	119.6 (3)
H21—O2W—H22	109.8	N1—C13—H13	120.2
Eu1—O3W—H31	120.1	C12—C13—H13	120.2
			
O3^i^—Eu1—O1—C1	−9.5 (3)	C4—C5—C6—C7	−2.2 (5)
O4^ii^—Eu1—O1—C1	144.7 (2)	C4—C5—C6—S1	177.6 (3)
O4W—Eu1—O1—C1	59.5 (2)	O5—S1—C6—C7	−109.9 (3)
O2W—Eu1—O1—C1	−160.0 (2)	O7—S1—C6—C7	12.3 (3)
O1W—Eu1—O1—C1	123.4 (2)	O6—S1—C6—C7	130.4 (3)
O3W—Eu1—O1—C1	−73.6 (2)	O5—S1—C6—C5	70.4 (3)
O5W—Eu1—O1—C1	−110.2 (2)	O7—S1—C6—C5	−167.5 (3)
O2—Eu1—O1—C1	2.17 (19)	O6—S1—C6—C5	−49.4 (3)
O3^i^—Eu1—O2—C1	168.6 (2)	C5—C6—C7—C2	2.0 (5)
O4^ii^—Eu1—O2—C1	−119.5 (2)	S1—C6—C7—C2	−177.8 (3)
O4W—Eu1—O2—C1	−119.1 (2)	C3—C2—C7—C6	−1.3 (5)
O2W—Eu1—O2—C1	18.3 (2)	C1—C2—C7—C6	177.3 (3)
O1W—Eu1—O2—C1	−56.4 (2)	Eu1^i^—O3—C8—O4	36.9 (12)
O3W—Eu1—O2—C1	82.5 (2)	Eu1^i^—O3—C8—C4	−142.6 (8)
O1—Eu1—O2—C1	−2.2 (2)	Eu1^iv^—O4—C8—O3	42.7 (7)
O5W—Eu1—O2—C1	111.6 (2)	Eu1^iv^—O4—C8—C4	−137.8 (3)
Eu1—O2—C1—O1	3.8 (3)	C5—C4—C8—O3	−169.6 (3)
Eu1—O2—C1—C2	−176.0 (3)	C3—C4—C8—O3	8.6 (5)
Eu1—O1—C1—O2	−4.2 (4)	C5—C4—C8—O4	10.9 (5)
Eu1—O1—C1—C2	175.7 (2)	C3—C4—C8—O4	−170.9 (3)
O2—C1—C2—C7	−167.3 (3)	O8—N1—C9—C10	−179.0 (3)
O1—C1—C2—C7	12.8 (5)	C13—N1—C9—C10	0.8 (6)
O2—C1—C2—C3	11.2 (5)	N1—C9—C10—C11	0.1 (6)
O1—C1—C2—C3	−168.6 (3)	C9—C10—C11—C12	−1.5 (6)
C7—C2—C3—C4	0.8 (5)	C9—C10—C11—C11^iii^	177.1 (4)
C1—C2—C3—C4	−177.7 (3)	C10—C11—C12—C13	2.2 (6)
C2—C3—C4—C5	−1.0 (5)	C11^iii^—C11—C12—C13	−176.5 (4)
C2—C3—C4—C8	−179.2 (3)	O8—N1—C13—C12	179.6 (3)
C3—C4—C5—C6	1.7 (5)	C9—N1—C13—C12	−0.1 (6)
C8—C4—C5—C6	179.9 (3)	C11—C12—C13—N1	−1.4 (6)

Symmetry codes: (i) −*x*+1, −*y*+1, −*z*; (ii) *x*−1, *y*+1, *z*; (iii) −*x*+1, −*y*+1, −*z*+1; (iv) *x*+1, *y*−1, *z*.

### Hydrogen-bond geometry (Å, °)

**Table:**

*D*—H···*A*	*D*—H	H···*A*	*D*···*A*	*D*—H···*A*
O1w—H11···O8	0.84	1.87	2.673 (4)	159
O1w—H12···O6w	0.84	1.91	2.710 (4)	160
O2w—H22···O1^v^	0.84	2.11	2.804 (4)	140
O2w—H21···O3w^v^	0.84	2.46	3.148 (5)	140
O3w—H31···O7w	0.84	1.91	2.742 (4)	175
O3w—H32···O8w	0.84	1.87	2.704 (4)	172
O4w—H41···O8^vi^	0.83	1.82	2.648 (4)	171
O4w—H42···O5^vii^	0.83	2.02	2.840 (4)	166
O5w—H51···O7w	0.83	2.30	3.026 (5)	146
O6w—H62···O6^viii^	0.85	2.18	2.955 (5)	151
O6w—H61···O6^ii^	0.85	2.18	2.807 (5)	130
O7w—H71···O1^v^	0.84	2.49	3.191 (4)	142
O7w—H72···O7^ix^	0.84	2.03	2.860 (5)	169
O8w—H81···O2^i^	0.85	2.17	2.867 (4)	140
O8w—H82···O5^x^	0.85	2.07	2.879 (5)	159

Symmetry codes: (v) −*x*+1, −*y*+2, −*z*; (vi) −*x*, −*y*+2, −*z*+1; (vii) *x*−1, *y*, *z*; (viii) −*x*+1, −*y*+2, −*z*+1; (ii) *x*−1, *y*+1, *z*; (ix) *x*, *y*, *z*−1; (i) −*x*+1, −*y*+1, −*z*; (x) −*x*+2, −*y*+1, −*z*.
